# A Fluorescence In Situ Hybridization (FISH) Test for Diagnosing Babesiosis

**DOI:** 10.3390/diagnostics10060377

**Published:** 2020-06-06

**Authors:** Jyotsna S. Shah, Olivia Mark, Eddie Caoili, Akhila Poruri, Richard I. Horowitz, Alan D. Ashbaugh, Ranjan Ramasamy

**Affiliations:** 1 ID-FISH Technology Inc., 556 Gibraltar Drive, Milpitas, CA 95035, USA; 2 IGenex Inc., 556 Gibraltar Drive, Milpitas, CA 95035, USA; omark@igenex.com (O.M.); ecaoili@igenex.com (E.C.); aporuri@igenex.com (A.P.); 3 Hudson Valley Healing Arts Center, New York, NY 12538, USA; kalachakra108@aol.com; 4 Member, HHS Subcommittee on Babesia and Tick-Borne Pathogens, US Department of Health and Human Services, Washington, DC 20201, USA; 5 College of Medicine, University of Cincinnati, Cincinnati, OH 45221, USA; ashbauad@ucmail.uc.edu

**Keywords:** *Babesia*, babesiosis, FISH, fluorescence in situ hybridization, laboratory diagnosis of babesiosis

## Abstract

Apicomplexan parasites of the genus *Babesia* cause babesiosis in humans and animals. The microscopic examination of stained blood smears, detection of serum antibodies by immunoassays, and PCR-based identification of parasite nucleic acid in blood are common laboratory methods for diagnosing babesiosis. The present study evaluated a commercially available *Babesia* genus-specific fluorescence in situ hybridization (FISH) test for detecting *Babesia* parasites in blood smears. The FISH test detected *Babesia duncani* and *Babesia microti*, two common species that cause human infections in the USA, and other *Babesia* species of human and veterinary importance in less than two hours. The *Babesia* genus-specific FISH test supplements other existing laboratory methods for diagnosing babesiosis and may be particularly useful in resource-limited laboratories.

## 1. Introduction

Protozoan parasites of the genus *Babesia* that cause babesiosis in humans and animals [1,2,3] led to 2358 confirmed human infections in the US in 2017 [4]. *Babesia* are apicomplexan parasites that replicate within red blood cells and are transmitted to humans by ticks that acquire infections from reservoir animals such as white-footed mice [3,4]. Babesiosis can be less frequently transmitted between persons by blood transfusion, congenital transmission, and organ transplantation [3,4,5]. Human babesiosis is principally caused by *Babesia microti*, *B. duncani*, and *B. divergens* in the US [2,3,4]. *Babesia microti* is more prevalent on the east coast and *B. duncani* on the west coast of North America [6,7,8]. *Babesia microti*, *B. divergens*, *B. venatorum*, and *B. crassa* cause babesiosis in Eurasia [2,3]. Human babesiosis is also prevalent in Africa, South America, and Australia [2,3]. Moreover, *Babesia* infect cattle (e.g., *B. bovis*, *B. divergens*, *B. bigemina*), horses (e.g., *B. caballi*), domestic pets (e.g., *B. canis* in dogs, *B. felis* in cats), deer (e.g., *B. odocolei*, *B. venatorum*), mice (e.g., *B. microti*, *B. rodhani*), and other animals [3]. *Ixodes scapularis*, *I. ricinus*, *I. persulcatus*, and *Dermacentor albipictus* are examples of hard ticks that transmit *Babesia* to humans [3,4,7].

Common laboratory tests for diagnosing babesiosis depend on detecting (i) parasites in stained blood smears by microscopy, (ii) serum antibodies by immunoassays, and (iii) *Babesia* nucleic acid in blood after PCR amplification [3,4]. Fluorescence in situ hybridization (FISH) tests are used for detecting malaria parasites in blood smears [9,10] and tuberculosis bacilli in sputum smears [11,12]. We now report the details of a commercially available FISH test designed to detect all known species of *Babesia* parasites in blood smears. The FISH test is based on DNA probes that hybridize to the multiple copies of 18S ribosomal RNA (rRNA) present in *Babesia* cells, and therefore the test does not entail PCR amplification of nucleic acid.

## 2. Materials and Methods

### 2.1. FISH Assay Reagents

The study utilized the *Babesia* genus-specific FISH assay kit (catalogue number BabGK04) available from ID-FISH Technology Inc. (Milpitas, CA, USA). The kit contained DNA probes targeting *Babesia* 18S rRNA, a hybridization buffer, a smear preparation reagent (SPR), necessary buffers, a counter stain, and a slide mounting medium. The DNA probes were covalently labeled with Alexa 488 green fluorescent dye.

### 2.2. FISH Test Method

The *Babesia* genus-specific test was performed according to the manufacturer’s instructions provided with the kit similar to the FISH test procedures for *Plasmodium falciparum* and *Plasmodium vivax* [9] and *Plasmodium knowlesi* [10]. Three parts EDTA-treated whole blood or fresh cultures for testing were first mixed with 1 part SPR by volume. A thin smear on a glass microscope slide prepared from 4 μL of this mixture was air-dried and fixed with methanol. After adding 12 μL of hybridization buffer containing the probe mix to each fixed smear, it was covered with a plastic cover-slip and placed in a humid container at 37 °C for 30 min for hybridization. After 30 min, the smear was washed twice for 2 min each with wash buffer at an ambient temperature, followed by a rinse with rinse buffer. A drop of counterstain was added after drying the smear in complete darkness. The smear was then covered with a glass cover-slip and viewed at ×1000 magnification in an Olympus light microscope (Olympus, Tokyo, Japan) with an attached LED unit containing 492 nm excitation and 530 nm emission band pass filters (ID-FISH, Milpitas, CA, USA). *Babesia* parasites fluoresce green under these conditions. 

### 2.3. Analytical Specificity of the Babesia Genus FISH Test

The FISH test was performed on smears prepared as described above from 12 pertinent species of bacteria and non-*Babesia* parasites in addition to *Babesia duncani* and *Babesia microti* grown in hamster blood as positive controls and a negative control of uninfected human blood (Table 1). An online BLASTn analysis at the National Centre for Biological Information (NCBI, Bethesda, MD, USA https://blast.ncbi.nlm.nih.gov) of the DNA probe sequences used in the *Babesia* genus FISH test was also performed against the human genome as well as Apicomplexa (taxid 5794), *Theileria* (taxid 5873), *Cytauxzoon* (taxid 27995), and *Babesia* (taxid 5864) genomes.

### 2.4. Detection of Different Babesia Species in the Babesia Genus-Specific FISH Test

The *Babesia* genus-specific FISH test was performed on thin blood smears prepared as described above from (i) hamster blood infected with *B. microti* and *B. duncani* (provided by Dr. Alan Ashbaugh, University of Cincinnati, OH, USA), (ii) an in vitro culture of *B. divergens* in human blood (Cat. No. CA-DIV-12, Fuller Laboratory, Fullerton, CA, USA) and (iii) bovine blood infected with *B. bigemina* and *B. bovis* (provided by Dr. Massaro Ueti, Washington State University, WA, USA). Blood smears from two patient blood samples received at IGeneX for routine testing and confirmed to be infected respectively with *B. duncani* and *B. microti* by DNA sequencing were also tested.

### 2.5. Analytical Sensitivity of the Babesia Genus FISH Test

The analytical sensitivity was measured as the limit of detection (LOD) as previously described for FISH tests on *P. falciparum*, *P. vivax*, and *P. knowlesi* [9,10]. The LOD was determined as the lowest concentration of parasites that could be detected by FISH in every one of 20 replicate smears prepared as described above at different serial dilutions of *B. duncani* (ATCC PRA-302) and *B. microti* (ATCC 30221D) grown in hamster blood provided by Dr. Alan Ashbaugh, University of Cincinnati, OH, USA. The starting parasitemia in hamster blood was determined from Giemsa-stained blood smears. The hamster blood was initially diluted to 1:1000 with uninfected human blood and then serially diluted two-fold in the same human blood to a final dilution of 1:32,000, before performing FISH tests on triplicate blood smears at each dilution. Once a preliminary LOD was determined, 17 additional smears made at the same dilution were tested for confirming the LOD.

### 2.6. Detection of B. duncani and B. microti in Clinical Blood Samples 

From clinical blood samples originating in different US states that were received at IGeneX for routine testing and found to be positive in the *Babesia* genus FISH test, 44 were randomly selected for DNA sequencing after PCR amplification to further confirm that the test can detect both *B. duncani* and *B. microti* in clinical samples. For this purpose, DNA was first purified from 200 μL of EDTA-treated whole blood using the Generation Capture Column kit (Qiagen, Valencia, CA, USA). The purified DNA was then PCR-amplified in an Eppendorf Thermocycler 7000 using forward and reverse primers (Integrated DNA Technologies, San Diego, CA, USA) that targeted a species-variable region of *Babesia* 18S rDNA to yield an approximately 350 bp amplicon. For each PCR reaction, 9 μL of DNA was combined with 21 μL of the PCR reaction mixture (10 mM Tris-HCl pH 8.3, 50 mM KCL, 5 mM MgCl2, 0.001% gelatin, 1% glycerol, 0.5 μg of each primer, 0.1 mM dNTPs, and 0.25 U Amplitaq Gold from Applied Biosystems, Foster City, CA, USA). The PCR conditions employed were an initial denaturation at 95 °C for 10 min, followed by 50 cycles using a denaturation at 95 °C for 1 min, annealing at 57 °C for 1 min, and extension at 72 °C for 1 min. A final 10 min extension was performed at 72 °C. The PCR product was subsequently sequenced in both directions using the same forward and reverse primers to provide a minimum of 200 contiguous bases with a PHRED quality value of 20 or higher. The resulting DNA sequence was matched to the NCBI reference sequences for *B. duncani* and *B. microti*, with a cut-off BLASTn E-value of < 10^−30^.

### 2.7. Estimation of Clinical Diagnostic Parameters of the Babesia Genus FISH Test

A set of 154 clinical blood samples were received by IGeneX from the New York State Department of Health (NYSDOH) for proficiency testing for the *Babesia* genus FISH test. All 154 samples had been previously tested at NYSDOH by examining Giemsa-stained blood smears for *Babesia* parasites, and 51 were found to be positive. All 154 samples were tested by the *Babesia* genus FISH test at IGeneX in a blinded manner without prior knowledge of the Giemsa stain results.

## 3. Results

### 3.1. Analytical Specificity

Among all the samples tested in Table 1, only *Theileria equi* in addition to *B. duncani* and *B. microti* reacted positively in the *Babesia* genus-specific FISH test. All the others were correctly identified as negative in the test. The online BLASTn analysis (NCBI, Bethesda, MD, USA) predicted that the *Babesia* genus-specific probes would detect 18S rRNA of *T. equi* but not 18S rRNA of other *Theileria* species or human DNA under the FISH test conditions. The BLASTn analysis also predicted that the *Babesia* genus probes would detect the 18S rRNA of all the animal and human *Babesia* parasite species in the NCBI database and may weakly detect *Cytauxzoon felis* 18S rRNA under the FISH test conditions.

### 3.2. Detection of Different Babesia Species

**Figure 1:**
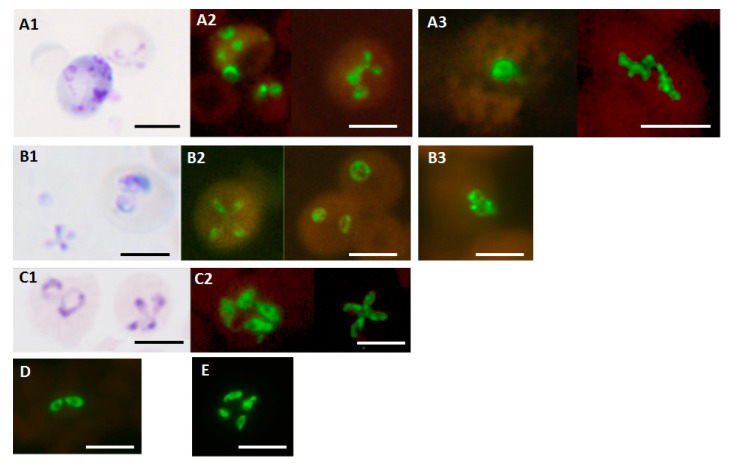
Photographs showing the results of the *Babesia* genus-specific FISH test on different *Babesia* species. A—*B. microti*; B—*B. duncani*; C—*B. divergens*; D—*B. bovis*; E—*B. bigemina* with parasite sources described in the Methods and Materials section. Photographs of the parasites stained with Giemsa in the smears used for the FISH tests are shown in the case of *B. microti* (**A1**), *B. duncani* (**B1**), and *B. divergens* (**C1**). Fluorescence observed in the corresponding FISH tests on smears from the same preparations are shown in (**A2**) for *B. microti*; (**B2**) for *B. duncani*; (**C2**) for *B. divergens*. Fluorescence observed in the FISH tests performed on *B. bovis* (**D**) and *B. bigemina* (**E**) are also shown. Fluorescence observed in the FISH tests performed on different blood smears from two patient samples received for testing at IGeneX with species confirmed by DNA sequencing are shown in (**A3**) for *B. microti* and (**B3**) for *B. duncani*. Photographs were taken at ×1000 magnification. Scale bars represent approximately 5 μm.

Photographs showing the results of the *Babesia* genus-specific FISH tests performed on blood smears containing *B. microti*, *B. duncani*, *B. divergens*, *B. bovis*, and *B. bigemina* are shown in Figure 1. They demonstrate that the *Babesia* genus-specific FISH test is able to detect the human parasites *B. microti* (Figure 1(A2,A3)) and *B. duncani* (Figure 1(B2,B3)) in hamster and patient blood as well as in vitro-cultured *B. divergens* (Figure 1(C2)). A1, B1, and C1 are respective photographs of Giemsa-stained smears of the *B. microti*, *B. duncani*, and *B. divergens* used in the FISH tests. In addition, the FISH assay is also able to detect *B. bovis* (Figure 1D) and *B. bigemina* (Figure 1E), the two important parasites causing bovine babesiosis. The characteristic tetrad of merozoites, giving the appearance of a Maltese cross [3], was present in the Giemsa-stained (Figure 1(A1,B1,C1)) and FISH-reactive (Figure 1(A2,A3,B2,C2)) *Babesia* smears.

### 3.3. Analytical Sensitivity

The greatest dilutions of the *Babesia*-infected hamster blood at which *B. duncani* and *B. microti* could be detected in the FISH test in every one of the replicate smears were 1:16,000 and 1:8000, respectively. Based on the parasitemia of 904,176 per μL for *B. duncani* and 232,541 per μL for *B. microti* in the undiluted hamster blood, this corresponded to a LOD of 57 *B. duncani* and 58 *B. microti* per μL of blood or an approximate parasitemia of 0.001% for both species.

### 3.4. Detection of B. duncani and B. microti in Clinical Blood Samples

DNA sequencing showed that of the 44 randomly selected US clinical blood samples that were positive in the *Babesia* genus FISH test, 21 were infected with *B. duncani* and 23 with *B. microti*. Coinfections with *B. duncani* and *B. microti* were not observed in the 44 samples.

### 3.5. Estimated Clinical Diagnostic Parameters of the Babesis Genus FISH Test

Of the 154 proficiency test clinical blood samples from NYSDOH, 50 were found to be positive in the *Babesia* genus FISH test. Only the same 50 samples and one additional sample (*n* = 51) had been independently found by the NYSDOH to possess *Babesia* parasites on examination of the Giemsa-stained blood smears of all 154 samples. The remaining 103 samples were negative by both Giemsa staining and *Babesia* genus FISH. The clinical parameters of the *Babesia* genus FISH test relative to Giemsa staining estimated from these findings are presented in Table 2.

## 4. Discussion

*Ixodes* ticks that transmit *Borrelia* species causing Lyme disease and Relapsing Fever can also transmit *Babesia* to humans [4,13,14]. Babesiosis and borreliosis can present with similar clinical manifestations and occur as coinfections in patients in the US [15,16,17,18,19]. The clinical symptoms of babesiosis also overlap with those of malaria [20]. The early intra-erythrocytic stages of human-infecting *Babesia* species in Giemsa or Wright’s stained blood smears can be mistaken for the ring and trophozoite stages of *P. falciparum*, and this can lead to misdiagnosis in localities where babesiosis and malaria are co-endemic [4]. The ability of the *Babesia* genus-specific FISH test to differentiate *Babesia* infections in blood from those caused by *Borrelia*, *Plasmodium* and other pertinent blood-borne pathogens is therefore important for diagnosing babesiosis.

Our findings suggest that the *Babesia* genus-specific FISH test is useful for diagnosing human infections with different *Babesia* species well as *B. bovis* and *B. bigemina* infections in cattle and infections with other *Babesia* species in animals [21]. The *Theileria* species infecting livestock, with the exception of *T. equi* in horses, are not expected to be positive in the *Babesia* genus-specific FISH test, but additional investigations are needed to firmly establish this. Because *Theileria equi* was until recently classified as *Babesia equi*, it may be phylogenetically closer to *Babesia* than other *Theileria* species, leading to the cross-reactivity observed in the *Babesia* genus-specific FISH test. *Cytauxzoon felis* is a tick-borne apicomplexan parasite that infects felids worldwide and causes severe disease among domestic cats in the US [22]. The ability of the *Babesia* genus-specific FISH test to differentiate *B. felis* from *C. felis* requires further investigation, because in silico analysis predicts that there may be a weak cross-recognition of *C. felis*.

Serum antibody assays are widely used for diagnosing human and veterinary babesiosis, but the detection of antibodies does not readily distinguish between active and resolved *Babesia* infections [1,2,3]. Several types of PCR-based nucleic acid amplification tests for detecting *Babesia* parasites in blood are reported to have a greater sensitivity [23,24] than our finding of 57–58 *Babesia* parasites per μL in the *Babesia* genus FISH test. Real-time PCR (RT-PCR) tests are therefore recommended for screening donor blood for babesiosis in endemic areas of the US [25,26]. FISH tests targeting rRNA discriminate between viable and dead or dying parasites better than PCR tests targeting DNA because RNA is degraded more rapidly than DNA in dying cells [9,27], a property that is useful for monitoring the effects of drug treatment. Cost and infrastructure requirements also make FISH tests more practical than PCR-based tests for resource-limited laboratories. A LED/Filter unit ($3100 from IDFISH, Milpitas, CA, USA), a standard microscope (e.g., Olympus BX41, $3000, from Olympus, Tokyo, Japan), a 37 °C incubator ($410), and reagents/disposables ($2.60 per test) are required with minimal maintenance costs for FISH. FISH can be performed on a normal laboratory bench. On the other hand, a RT-PCR machine with analytical software ($22,500), two biosafety hoods ($6000), annual maintenance ($3800), and reagent/disposables ($3.50 per test) are estimated to be more costly. RT-PCR also requires a dedicated work area. 

The *Babesia* genus FISH test allows the direct visualization of the important human parasites causing babesiosis in the US, viz. *B. microti*, *B. duncani* and *B. divergens* [1,2,3,4], as well as the bovine parasites *B. bigemina* and *B. bovis* [21]. In silico analysis suggests that the test will also detect other known species of *Babesia* that infect humans and animals worldwide. The FISH test reproducibly detects parasitemias as low as 57–58 *Babesia* parasites per μL or about 0.001%, and showed congruent results with a qPCR test performed independently in a different laboratory [24]. The analytical sensitivities in the *Babesia* genus FISH test for *B. duncani* and *B. microti* are comparable to those in FISH tests for *P. falciparum* (55–62 parasites per μL), *P. vivax* (56–59 parasites per μL), and *P. knowlesi* (61–84 parasites per μL), which were better than the examination of the corresponding Giemsa-stained blood smears [9,10].

Results with clinical samples estimate a clinical sensitivity, specificity, positive predictive value, and negative predictive value of 98%, 100%, 100%, and 99% respectively for the *Babesia* genus FISH test in comparison to independent detection by Giemsa staining. It is possible that the one sample positive by Giemsa staining at the NYSDOH but not by the FISH test at IGeneX may have been due to the presence of dead or dying parasites as a result of immune damage or drug treatment, but this could also have been a missed detection of low numbers of live parasites by FISH. Investigations into many different types of well-characterized clinical samples are needed to further establish the clinical diagnostic parameters of the *Babesia* genus FISH test.

The *Babesia* genus FISH test, like the similar FISH tests for malaria [9,10] and tuberculosis [11,12], provides a result in less than 2 h, detects only live parasites, requires minimal technical expertise and equipment, needs only an LED/filter unit attached to a standard light microscope for visualizing fluorescence, and utilizes stable reagents that do not require refrigeration. As nucleic acid amplification by PCR is not involved, the FISH test is not affected by interference from PCR inhibitors present in some clinical samples [28]. FISH using oligodeoxynucleotides to target rRNA was first shown in 1989 to distinguish closely related bacteria with appropriately-targeted probes labeled with different fluorescent dyes [29]. The development of species-specific FISH tests to distinguish different *Babesia species* in the manner shown possible for differentiating pathogen species in FISH tests for malaria [9,10] and tuberculosis [11,12] will be useful. Preliminary findings suggest that this may be possible for *Babesia venatorum* in China [30] as well as *B. microti* and *B. duncani* in the USA [31], but further investigations are required to confirm the species specificity of the respective probes.

## 5. Conclusions

The *Babesia* genus-specific FISH test has advantages that make it a useful supplement to the panel of tests currently available for the laboratory diagnosis of human babesiosis as well as babesiosis in pets and livestock.

## Figures and Tables

**Table 1:** Reference pathogens tested in the *Babesia* genus fluorescence in situ hybridization (FISH) test.

Bacteria	Source
*Anaplasma phagocytophilum*	Patient blood from IGeneX positive for *Anaplasma phagocytophilum*
*Bartonella henselae*	ATCC 49882 in vitro culture
*Borrelia burgdorferi*	ATCC 35210-B31 in vitro culture
*Borrelia hermsii*	DSM 5251 in vitro culture
*Ehrlichia chaffeensis*	Patient blood from IGeneX positive for *Ehrlichia chaffeensis*
**Protozoa**	
*Leishmania donovani*	ATCC 50212 in vitro culture
*Plasmodium falciparum*	Patient blood from Kenya
*Plasmodium malariae*	Patient blood from Kenya
*Plasmodium ovale*	Patient blood from Kenya
*Plasmodium vivax*	Patient blood from India
*Theileria equi*	BEG-120 in vitro culture in equine blood, Fuller Lab, Fullerton, CA
*Trypanosoma cruzi*	ATCC 50823 in vitro culture
**Controls**	
Negative control	Uninfected human blood
Positive control 1	Hamster blood infected with *Babesia duncani* ATCC PRA-302
Positive control 2	Hamster blood infected with *Babesia microti* ATCC 30221D

*Anaplasma phagocytophilum* and *Ehrlichia chaffeensis* infections in patients were confirmed by immunofluorescence assays at IGeneX, as described at www.igenex.com. Hamster blood infected with *Babesia duncani* and *Babesia microti* were provided by Dr. Alan Ashbaugh, University of Cincinnati, OH, USA.

**Table 2:** Estimated clinical diagnostic parameters for the *Babesia* genus FISH test.

FISH	Giemsa		Clinical Diagnostic Parameter	Estimate (95% CI)
	(+)	(−)	Sensitivity	98% (88–100)
(+)	50	0	Specificity	100% (96–100)
(−)	1	103	Positive Predictive Value	100% (91–100)
Total	51	103	Negative Predictive Value	99% (94–100)

CI—confidence interval.
